# Validation of modified COVID-19 Phobia Scale (MC19P-SE) to examine the relationships between corona anxiety and COVID-19 symptoms: A case-control study

**DOI:** 10.1016/j.xjmad.2025.100108

**Published:** 2025-01-16

**Authors:** Luthful Alahi Kawsar, Syed Toukir Ahmed Noor, Md. Atiqul Islam, Mohammad Romel Bhuia

**Affiliations:** a Department of Statistics, Shahjalal University of Science & Technology, Sylhet 3114, Bangladesh; b Department of Statistics, Jagannath University, Dhaka, Bangladesh

**Keywords:** COVID-19, Coronaphobia, COVID-19 related anxiety, COVID-19 symptoms, Bangladesh

## Abstract

**Purpose:**

The main purpose of the present study is to validate the modified COVID-19 phobia scale (MC19P-SE) in Bangla and use it to identify the link between COVID-related anxiety and the onset of COVID-19 symptoms.

**Methods:**

A retrospective case-control study was conducted in Sylhet, a northeastern district in Bangladesh, focusing on a population of 18 years or older. The MC19P-SE scale was used to gather data on coronaphobia among the respondents, and factor analysis was used to derive reliable factors. We employed a multivariable logistic regression model to identify the relationships between coronavirus anxiety and COVID-19 symptoms.

**Results:**

Among the 482 participants, 42 % manifested COVID-19 symptoms. Factor analysis revealed four underlying factors: psychological anxiety, psychosomatic anxiety, economic anxiety, and social or excessive protective anxiety. MC19P-SE exhibited reliability with a Cronbach alpha coefficient of 0.91 for full-scale. According to the model, psychosomatic anxiety increased (AOR= 1.14, 95 % CI: 1.07, 1.22) the risk while social or excessive protective anxiety decreased (AOR=0.88, 95 % CI: 0.78, 0.99) the risk of developing COVID-19 symptoms significantly.

**Conclusion:**

The research found a significant relation in the Bangladeshi district of Sylhet between the onset of COVID-19 symptoms and anxiety associated with the virus. Furthermore, the findings affirm the potential reliability and validity of the MC19P-SE scale, strengthening its efficacy for future research and evaluations.

## Introduction

Anxiety is a psychological condition characterized by exaggerated ongoing fear, frequently accompanied by physical symptoms [1]. Although anxiety symptoms may vary, they often involve nervousness, restlessness, stress, constant worry, upset stomach, feeling overwhelmed, and physical signs such as tense muscles and a fast heartbeat [2]. In addition to increasing morbidity and mortality, anxiety can aggravate the symptoms of other diseases and is the excessive and illogical fear of having a severe health condition [3]. Research showed that health anxiety may contribute to heightened perceptions of the virus's dangerousness [4]. Also, factors that have been common during the epidemic, such as social isolation and economic concerns, might lead to greater anxiety [5]. The COVID-19 epidemic greatly affected daily living and had led to various psychological issues among the general population, including sleep disturbances, depression, panic attacks, and fear of contracting the disease [6], [7]. Mental Health America (MHA) reported that by the end of 2021, compared to pre-COVID-19 time, 5.4 million more people were suffering from moderate to severe depression [8]. Daily anxiety sufferers may reach a level of panic that results in symptoms similar to those of COVID-19 [9].

The term "coronaphobia" or "corona anxiety" was first used because of the widespread psychological effects of COVID-19 [10]. Corona anxiety is associated with disrupting daily routines, misinterpreting mild symptoms, and putting yourself at risk for social insecurity. These factors are thought to be caused by unexpected events, limitless uncertainty, the acceptance of new behavioral patterns, misbelief in health infrastructures, warnings from international organizations, and infodemics [11]. According to Taylor (2019), several emotional exposure factors, such as the lack of willingness to tolerate uncertainty, recognized exposure to disease, and anxiety, can play a role in coronaphobia [12]. The risk of getting infected by the virus or dying and the insufficient medical facilities at the national level make vulnerable individuals extremely stressed and generate serious psychological problems [13], [14], [15].

Previous research on different infectious diseases showed that quarantine caused psychological disorders such as posttraumatic stress symptoms, nervousness, disorientation, and anger [16], [17], [18], [19]. During the H1N1 influenza outbreak in 2009, approximately 10 %-30 % of the UK population was worried about getting infected, and during the SARS epidemic, indications of post-traumatic stress disorder and depression were found in 15 % of the quarantined people in Toronto [19]. Another study revealed that 25 % of the Indian people were suffering from anxiety due to the measures taken to control COVID-19 [20]. Uncertainty about the health risks of your loved one and yours, interference with routine life, and unfamiliar characteristics of the disease are the leading causes of anxiety [21]. A study conducted in Bangladesh revealed that approximately 85.60 % of the participants experienced sleeplessness, irritability, and disorder at home as a result of stress associated with COVID-19 [22]. Also, a few other studies on the Chinese population addressed the negative psychological impact of COVID-19 [23], [24], [25]. Furthermore, Daniali, H., and Flaten, M. A. conducted two relevant studies in Norway, exploring the phenomenon termed the 'nocebo effect.' This idea explores the psychological factors that cause people to believe they have COVID-19 even when they are not exhibiting the disease's symptoms. It offers important insights into the relationship between psychological factors and the perception of COVID-19 symptoms [26], [27].

In response to these contextual issues, Arpaci et al. [28] created and evaluated the COVID-19 Phobia Scale (C19P-SE), which enables researchers to evaluate the degree of COVID-19 fear among residents of different countries and communities. For this study, we adapted and translated the C19P-SE scale. Therefore, we have developed a modified version of the COVID-19 phobia scale (MC19P-SE) for use in the Bangladeshi population and evaluated the validity and reliability of the modified scale. Furthermore, while various studies have looked at the detrimental psychological influence of COVID-19 on people's lives, nothing is known about whether COVID-related anxiety or fear might contribute to psychological experiences of having COVID-19 symptoms [29], [30]. To our knowledge, no studies have looked at the potential connection between the onset of COVID-19 symptoms and virus-related anxiety. Thus, this is the first study to look at the connection between corona anxiety and COVID-19 symptoms.

## Methods

This study is presented in accordance with the Strengthening the Reporting of Observational Studies in Epidemiology (STROBE) declaration [31].

### Primary data: study design and sample size

#### Study design, setting, and participants

A pre-pilot questionnaire was designed to perform retrospective case-control research in order to gather primary data through interviews. Data were collected by simple random sampling from two groups of people: (i) patients who developed self-declared symptoms of COVID-19 (case group) and (ii) the general population who did not develop any symptoms of COVID-19 (control group) in Sylhet, a north-eastern district of Bangladesh. Only the adult population was included in this study, i.e., participants had to be at least 18 years old. Subjects who had other psychological illnesses (e.g., personality disorders) were excluded. Samples were gathered through in-person interviews between February and May of 2022.

The list of patients who developed COVID-19 symptoms (case group) was collected from the civil surgeon's office in Sylhet. The sampling frame of the control group was collected from the list of households in Sylhet, ensuring a random selection of individuals who reported no symptoms of COVID-19 during the pandemic.

#### Sample size calculation

Considering an expected 25 % prevalence of COVID-related anxiety with a desired precision of 5%, the study's minimal necessary sample size has been determined using the formula below [20].

$$n=\frac{z^{2}p_{\exp}(1-p_{\exp})}{d^{2}}$$

where,

n=required sample size

z=Critical value,for95%confidence interval,z=1.96

$p_{\exp}=\mathrm{Expected prevalence}=0.25$

d=desired absolute precision=0.05

Therefore,

$$n=\frac{1.96^{2}*0.25*0.75}{0.05^{2}}≅288.$$

However, to account for potential non-responses or dropouts, we oversampled and selected 500 participants. The final sample size was 482 respondents, resulting in a 96.4 % response rate from the selected 500 participants. This included 201 individuals from the case group and 281 from the control group, which was sufficient to ensure statistical power for the analyses performed [32], [33].

*Instruments: MC19P-SE (Modified COVID-19 Phobia Scale / COVID-19 Related Anxiety):*

Numerous instruments have been created to quantify the level of anxiety linked to COVID-19. For instance, Arpaci et al. [28] developed a 20-item COVID-19 Phobia Scale (C19P-SE), Lee [34] developed a 5-item Coronavirus Anxiety Scale (CAS), Şimşir et al. [35] created a 7-item Fear of COVID-19 Scale (FC-19S), and Taylor et al. [36] developed COVID Stress Scales (CSS) which has 36 items. All of these tools have adequate reliability and validity. Among these tools, C19P-SE claimed to judge phobic responses as it is developed according to the characterization and detection of phobia in the Diagnostic and Statistical Manual of Mental Disorders (DSM-5) [37]

Therefore, the modified COVID-19 Phobia Scale in Bangla was used to collect data on this study's respondents' coronaphobia levels (MC19P-SE). The 23 items on the self-reported MC19P-SE questionnaire each have a 5-point rating scale that indicates how anxious a person was due to the COVID-19 pandemic. Items were scored on a Likert scale ranging from "strongly disagree" to "strongly agree" (1−5). We also added three additional questions to capture extra-protective behaviors during the pandemic period, which were not fully represented in the original 20-item C19P-SE. These additional items included:

1. After the onset of the coronavirus pandemic, I was very anxious to go outside of the home.
2. After the onset of the coronavirus pandemic, I used extra protection (e.g., PPE, hand gloves, face shield, etc.) when going outside.
3. After the onset of the coronavirus pandemic, I avoided public gatherings (e.g., mosques, shopping malls, crowded places, markets, etc.).

The inclusion of these extra questions aimed to better reflect specific behaviors related to COVID-19 anxiety in the Bangladeshi context. The final score was determined by totaling each response, which ranged from 23 to 115, with higher scores indicating higher levels of coronaphobia.

### Study variables

#### Outcome variable

The outcome variable in our study was the development of one or more World Health Organization (WHO) suggestive symptoms of COVID-19, categorized as either "yes" or "no." COVID-19 symptoms, as defined by WHO, include fever, cough, shortness of breath, sore throat, fatigue, loss of taste or smell, muscle or body aches, headache, chills, congestion or runny nose, nausea or vomiting, and diarrhoea. Respondents were asked whether they had experienced any of these symptoms during the COVID-19 period in Bangladesh (March 2020 to August 2021), allowing us to classify their symptom status accordingly [38]. The response was categorized as "0" if the responder had not experienced any COVID-19 symptoms and as "1" if they had.

#### Covariates

Based on a review of existing literature, this study included variables that were identified as potentially related and assumed to be associated with the development of symptoms of COVID-19. These variables are distributed within several broad factors, such as socio-demographic factors (i.e., gender, education, occupation, age, religion, the current status of marriage, nature of the family, monthly household income, area of residence, the status of chronic disease) and factors related to COVID-19 phobia (i.e., psychological anxiety, psychosomatic anxiety, economic anxiety, social/excessive protective anxiety).

### Statistical analysis

Basic descriptive statistics (frequency, percentages, mean, median, and standard deviation (SD)) and graphical representation were used to describe the data. The relationship between the outcome variable and the categorical variables was examined using the chi-squared test.

In order to reduce the 23 items of the MC19P-SE items to a small number of underlying factors, this study also used exploratory factor analysis (EFA). This study also made use of the varimax rotation. We used the scree plot and factors with eigenvalues greater than one to extract the desired component. Furthermore, the sample adequacy of EFA was assessed using the Bartlett sphericity test and Kaiser-Meyer-Olkin (KMO) method, and the reliability of MC19P-SE was confirmed using Cronbach's α [39]. After that, utilizing 23 items from the MC19P-SE, confirmatory factor analysis (CFA) was employed to build the trustworthy underlying components. The model's fit to the data was assessed using a variety of metrics, including the Tucker-Lewis fit index (TLI), comparative fit index (CFI), normed fit index (NFI), incremental fit index (IFI), goodness-of-fit index (GFI), adjusted goodness-of-fit index (AGFI), root mean squared error of approximation (RMSEA), etc. [40]. We used “lavaan” and “semTools” packages to conduct CFA and “psych” package was used to conduct EFA in R version 4.3.0.

A multivariable logistic regression model was utilized to identify risk factors for COVID-19 symptoms. The model with the lowest Akaike information criterion (AIC) was finally determined to be the most optimum model after we used the backward selection strategy for both modeling and variable selection [41]. Furthermore, the Hosmer and Lemeshow goodness-of-fit tests were conducted to assess the model fitting, and the multicollinearity of the variables was investigated using Variance Inflation Factors (VIFs) (min. =1.22, max. = 3.05, and avg. = 2.39) [42]. The adjusted odds ratio (AOR) with a 95 % CI was used to determine the strength of the associations and a p-value < 0.05 was considered as statistical significance.

#### Ethical approval

In this study, authors assert that all procedures contributing to this work comply with the ethical standards of the relevant national and institutional committees on human experimentation and with the Helsinki Declaration of 1975, as revised in 2008 [43]. All procedures involving human subjects were approved by the research center of the Shahjalal University of Science and Technology (SUST), Sylhet-3114, Bangladesh (SUST Research Centre, approval code: PS/2021/1/32). First, the collected data were de-identified. Then, the anonymized data were stored and analyzed on a password-protected computer. An experienced interpreter first wrote the survey questionnaire in English before translating it into Bangla. The questionnaire was tested with a small sample of participants and researchers to confirm that the questions were appropriate and to make any necessary grammatical corrections. Before beginning the data collection, each participant’s verbal consent was witnessed and formally recorded.

## Results

### Descriptive statistics of study variables

In total, 482 respondents participated in the study, 57.68 % of whom were men. The participants' ages varied from 18 to 100 years old, with an average age of 31.55 years (SD = 12.94). Of them, 54.81 % had finished their higher education. Approximately 41 % of the respondents were students or unemployed, followed by government or private services (28 %). More than half of the participants were never married (51.56 %) and belonged to a nuclear family (55.77 %), and one-third (31 %) had a household income of BDT 15,000–30,000. Additionally, 17.84 % of the participants had chronic illnesses, and almost 77 % of the participants lived in cities. Of the respondents, 41.70 % had developed COVID-19 symptoms, and 31.54 % went to test if they were infected (Table 1).

**Table 1:** Distribution of respondents by study variables.

Variables	Categories	Frequency	Percentage (%)
**Gender**	Female	204	42.32
	Male	278	57.68
	Total	482	100.00
**Education level**	Secondary or lower	113	23.64
	Higher Secondary	103	21.55
	Higher	262	54.81
	Total	478	100.00
**Occupation**	Govt. Employee/ Private service	133	27.71
	Business / Self-employed / Housewife / Farmer or others	150	31.25
	Student/ Unemployed	197	41.04
	Total	480	100.00
**Religion**	Islam	415	87.18
	Hindu	61	12.82
	Total	476	100.00
**Marital Status**	Never Married	248	51.56
	Ever Married	233	48.44
	Total	481	100.00
**Family nature**	Nuclear	266	55.77
	Joint / Extended	211	44.33
	Total	477	100.00
**Household’s Income**	0–15,000	60	13.27
	15,000–30,000	140	30.97
	30,000–50,000	133	29.42
	50,000 +	119	26.33
	Total	452	100.00
**Area of residence**	Rural	113	23.54
	Urban	367	76.46
	Total	480	100.00
**COVID−19 symptoms**	No	281	58.30
	Yes	201	41.70
	Total	482	100.00
**COVID−19 test**	No	330	68.46
	Yes	152	31.54
	Total	482	100.00
**Chronic diseases status**	No	396	82.16
	Yes	86	17.84
	Total	482	100.00
**Age (mean ± SD)**	467	31.55 ± 12.93	
**Household Member (Median (IQR))**	443	5.00 (41.00)	

### Internal consistency and score of MC19P-SE

**Fig. 1:**
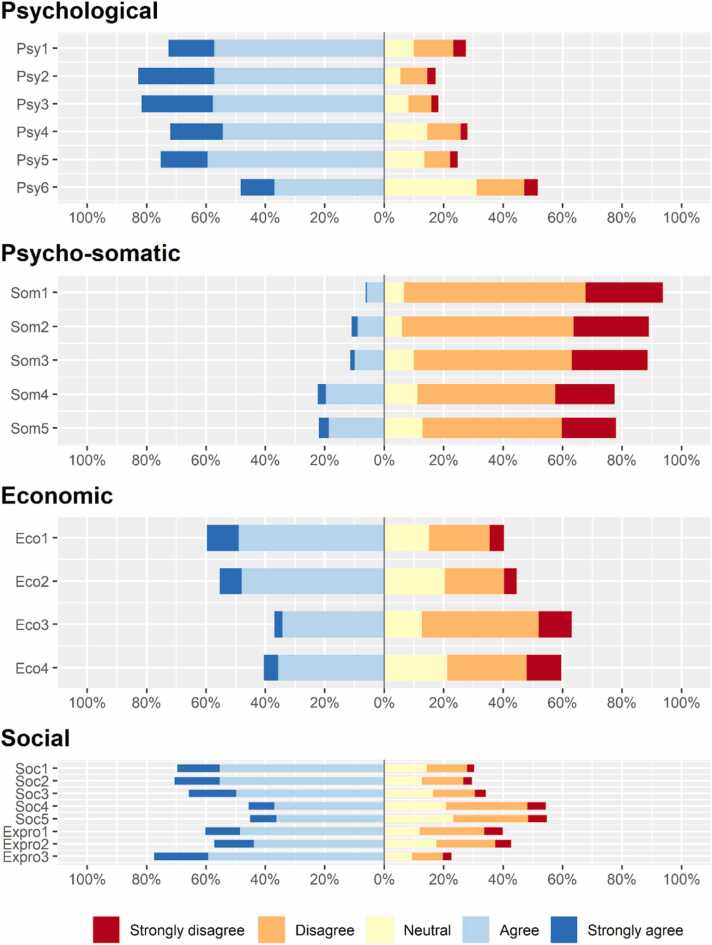
Distribution of participants’ responses for different factors.

Normality tests indicated that the kurtosis and skewness coefficients were below the ± 3 threshold, suggesting that the data had a normal distribution. The subscale reliability ranged from 0.76 to 0.85, whereas the full-scale Cronbach's alpha value was 0.905 (Table 2). Furthermore, in this study, the total phobia score ranged from 24 to 107 (mean=73.54, SD= 13.61). In terms of psychological anxiety, the maximum number of respondents (around 70 %) agreed with the questions, whereas they disagreed to higher degrees with psychosomatic anxiety. Furthermore, for economic-related anxiety, participants were almost neutral (they agreed more on two items and disagreed more on the remaining two items), and for social/excessive protective anxiety, they agreed more with the majority of the items (Fig. 1). The factors were significantly correlated with each other (p < .05) (Table 2).

**Table 2:** Inter-factor correlations, descriptive statistics, and reliability.

	**Psychological**	**Psycho-somatic**	**Economic**	**Social/excessive protective**
Psychological				
Psycho-somatic	0.322*			
Economic	0.335*	0.415*		
Social/excessive protective	0.601*	0.396*	0.502*	
Cronbach’s alpha **(0.905)**	0.851	0.853	0.757	0.842
Mean	22.39 (out of 30)	10.87 (out of 25)	12.48 (out of 20)	27.79 (out of 40)
Standard deviation	4.38	3.85	3.3	5.79

* p-value < 0.05.

### Exploratory factor analysis (EFA)

**Fig. 2:**
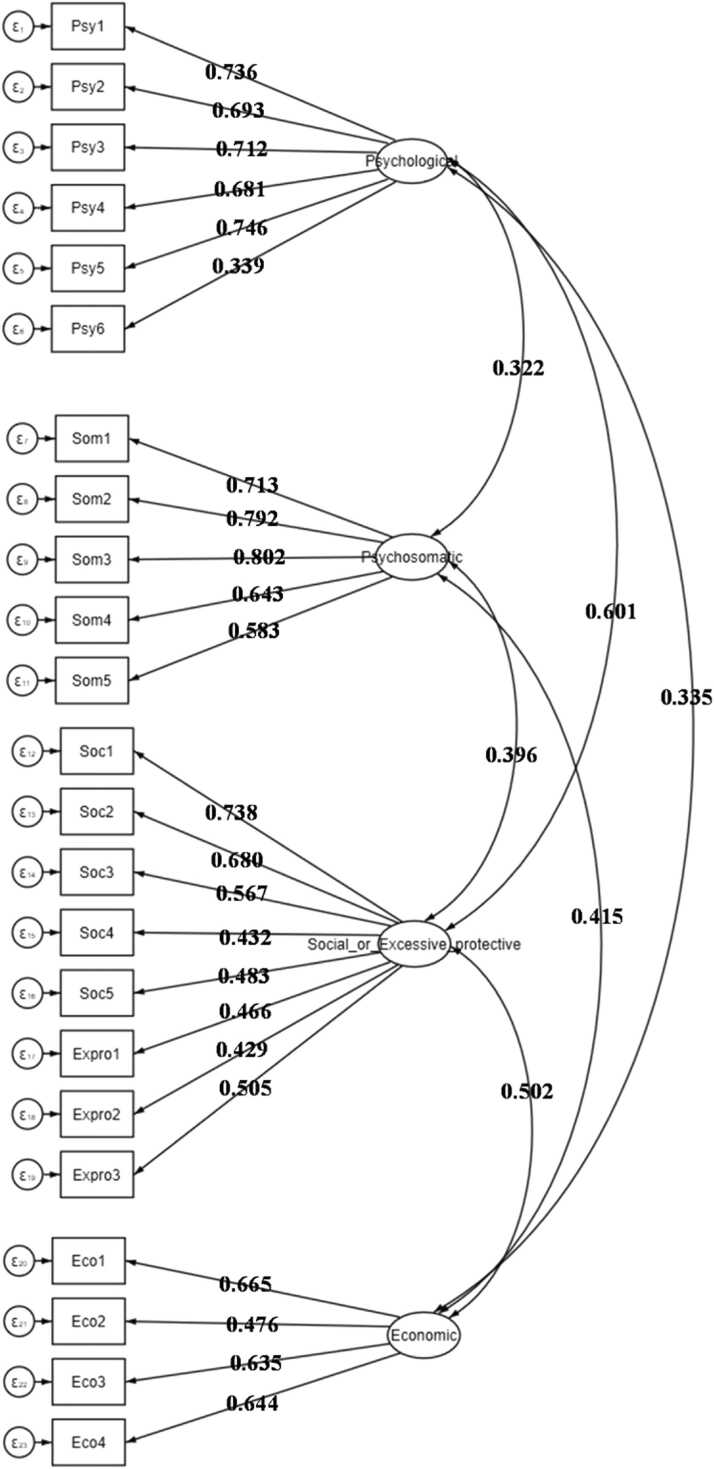
Item distribution, factor scores and inter-factor correlations.

In this study, the primary axis factoring approach has been used to conduct EFA. The KMO measure of sampling adequacy for the EFA was 0.90. Also, we found a significant Bartlett test of sphericity, χ2 (d.f=253) = 5038.767, p < 0.001. Based on the scree plot, we extracted four common factors (eigenvalue >1) that contained most of the information from the 23 items in MC19P-SE. Therefore, these four factors were sufficient to study the relation of the 23 items of MC19P-SE (Appendix Fig. 1**)**. So, we arrived at a four-factor solution (Fig. 2) where all commonalities were above the 0.30 cut-off mark (Appendix Table 1). A total of 57.74 % variation can be explained by selected four factors, which is divided as 16.09 % goes to the first component, 15.56 % to the second, 14.68 % to the third, and 11.43 % to the fourth. The heatmap of the correlation between factors and the clustering dendrogram is illustrated in Appendix Fig. 2.

### Convergent and discriminant validity

Convergence validity was assessed using composite reliability (CR) and average variance extracted (AVE) values. The findings demonstrate that CR is greater than the corresponding criteria of 0.70, but the AVE of the third and fourth factors could not suppress the threshold of 0.50. Furthermore, the square roots of all AVE values presented in the off-diagonal of Table 3 were greater than the corresponding cross-correlations, confirming the discriminant validity of the MC19P-SE. Along with the coefficients for convergence and discriminant validity, these findings indicate that MC19P-SE is a reliable and valid instrument for assessing anxiety related to COVID-19, Table 3 also includes interitem correlations.

**Table 3:** Convergent and discriminant validity.

**Factors**	**Composite Reliability (CR)**	**Average Variance Extracted (AVE)**	**Psychological**	**Psycho-somatic**	**Economic**	**Social/excessive protective**
Psychological	0.861	0.509	0.714*			
Psycho-somatic	0.857	0.549	0.349	0.741*		
Economic	0.759	0.447	0.419	0.517	0.668*	
Social/excessive protective	0.847	0.406	0.692	0.424	0.616	0.637*

### Construct validity

We performed a CFA in the research to evaluate how well the data fit the suggested four-factor structure. The fit indices for the CFA model of the MC19P-SE items are indicative of a reasonably good model fit. The GFI stands at 0.955, and the AGFI at 0.922, suggesting a relatively strong fit. Both CFI and IFI yield values of 0.969 and 0.970, respectively, indicating a good fit. The NFI and TLI at 0.932 and 0.952, respectively, also support the overall model fit. Additionally, the RMSEA with a value of 0.077 (95 % CI: 0.072–0.082) further reinforces the model's adequacy. Overall, it was clear that the model fit was adequate. (Table 4). Further, Appendix Fig. 3 shows how the items are embedded in each other, as well as latent variables, variances, and covariances (Appendix Fig. 3**)**.

**Table 4:** Fit indices for the CFA model of the MC19P-SE items.

Criteria	Value
Goodness of Fit Index (GFI)	0.955
Adjusted Goodness of Fit (AGFI)	0.922
Comparative Fit Index (CFI)	0.969
Normed Fit Index (NFI)	0.932
Incremental Fit Index (IFI)	0.970
Tucker-Lewis Fit Index (TLI)	0.952
Root Mean Squared Error of Approximation (RMSEA) (95 % CI)	0.077 (95 % CI: 0.072,0.082)

### Predictors of developing COVID-19 symptoms

The development of COVID-19 symptoms was significantly predicted by education level, employment, chronic disease status, place of residence, psychosomatic factors, and social/ excess protective factors. In terms of COVID-19 anxiety factors, the psychosomatic factor demonstrated a significantly higher risk (AOR = 1.14, 95 % CI: 1.07–1.22) for the development of COVID-19 symptoms. Meanwhile, the social/excessive protective factors exhibited a significantly lower effect (AOR = 0.88, 95 % CI: 0.78–0.99) in this regard. Compared to respondents with secondary or lower-level education, participants with higher secondary level were twice (AOR = 2.18, 95 % CI 1.06–4.51), and higher education were three times (AOR= 3.18, 95 % CI 1.60–6.32) more likely to have symptoms of COVID-19. Students or unemployed people were 94 % more likely (AOR= 1.94, 95 % CI: 1.01–3.73) to develop symptoms of COVID-19 than government employees or holders of private service. Individuals with chronic diseases had an almost two times higher likelihood of experiencing COVID-19 symptoms (AOR= 1.89, 95 % CI: 1.08–3.30) compared to those without chronic conditions. Additionally, people who lived in urban areas had an 89 % higher (AOR = 1.89, 95 % CI: 1.09–3.28) chance of having symptoms of COVID-19 compared to those who lived in rural regions (Table 5).

**Table 5:** Factor associated with developing the symptoms of COVID-19 in the multivariable logistic regression model.

**Variables**	**AOR (95 % CI)**	**P-value**
**Psychological anxiety/factor**	0.97 (0.92–1.03)	0.371
**Psycho-somatic anxiety/factor**	1.14 (1.07–1.22)	< 0.001*
**Economic anxiety**	0.98 (0.91–1.07)	0.702
**Social/excessive protective anxiety**	0.88 (0.78–0.99)	0.037*
**Education level**		
Secondary or lower^r^	1	
Higher Secondary	2.18 (1.06–4.51)	0.035*
Higher	3.18 (1.6–6.32)	0.001*
**Occupation**		
Govt. employee/ Private service^r^	1	
Business / Self-employed / Housewife / Farmer or others	0.95 (0.49–1.84)	0.876
Student/ Unemployed	1.94 (1.01–3.73)	0.047*
**Area of residence**		
Rural^r^	1	
Urban	1.89 (1.09–3.28)	0.024*
**Religion**		
Islam^r^	1	
Hindu	1.81 (0.96–3.43)	0.067
**Marital Status**		
Never married	1	
Ever Married	0.9 (0.47–1.7)	0.737
**Household’s Income**		
0–15,000^r^	1	
15,000–30,000	1.62 (0.79–3.32)	0.187
30,000–50,000	1.61 (0.78–3.33)	0.2
50,000 +	2.11 (0.99–4.53)	0.055
**Chronic disease status**		
No	1	
Yes	1.89 (1.08–3.3)	0.026*

The superscript “r” denotes the reference category in every case.

* p-value < 0.05

## Discussion

In this study, we conducted a factor analysis on 23 items of the MC19P-SE and finally came up with a result of four common factors named: psychological, psychosomatic, economic, and social/excessive protective anxiety. According to this study, there is a significant association between the development of COVID-19 symptoms and anxiety about COVID-19. Furthermore, we found some background characteristics that were also responsible for developing symptoms; these were education level, occupation, area of residence, and presence of chronic diseases. Among all COVID-related anxieties, psychosomatic and social/excessive protective anxiety showed a significant impact on COVID-19 symptoms.

In this study, MC19P-SE was found to be a reliable scale (23 items) with a Cronbach’s α of 0.91, and the reliability of the subscale varied from 0.76 to 0.85. Previous studies done in many countries utilizing translated versions of the scale further confirmed the measure's validity and reliability with a Cronbach's α range of 0.88–0.93 [44], [45], [46], [47]. Furthermore, the convergent validity of MC19P-SE was demonstrated since an AVE below 0.50 may be considered if the CR is greater than 0.70 [48].

An engrossing result unfolded by this current study is that there is a positive and negative impact of COVID-19 anxiety on developing the symptoms of COVID-19. In terms of psychosomatic factors, the development of symptoms had a positive impact. Participants who went through this anxiety tended to develop COVID-19 symptoms more. Psychosomatic anxiety was found to be responsible for the development and effect of COVID-19 in many types of literature, especially one study from Turkey [49]. Conversely, this study revealed that social/excessive protective anxiety was negatively correlated with the likelihood of experiencing symptoms of COVID-19, as it acted as a protective factor. Participants who exhibited high levels of caution and awareness of the SARS-CoV-2 virus were less likely to develop symptoms of COVID. Centers for Disease Control (CDC) and many other researchers have also suggested that proper protection is needed to avoid COVID-19 [50], [51], [52]. However, the study did not find any significant relationship of psychological and financial anxiety with COVID-19 symptoms. As participants in the study are relatively evenly distributed across various income levels, it might reduce the ability to observe significant associations between economic anxiety and COVID-19 symptoms. Even though Fig. 2 clearly showed that the majority of respondents experienced psychological distress throughout the pandemic, this did not have an impact on the emergence of COVID-19 symptoms. Few studies have found the opposite result, indicating that psychological distress and financial anxiety were significant predictors of health outcomes, including susceptibility to infections such as COVID-19, possibly due to stress-induced immune dysregulation and limited access to healthcare resources among financially strained individuals [53], [54], [55]. Furthermore, the existing studies emphasize the complex interaction of several variables in determining the association between financial and psychological distress and COVID-19 symptoms. Several factors, including gender, age, social support, and prior anxiety disorders, have been shown to moderate the impacts of psychological and financial anxiety, according to earlier research [56], [57]. The complexities of mental health dynamics during a global health crisis can be better understood by taking into account individual variations and environmental circumstances, as these nuanced findings highlight. Moreover, Kim's (2021) findings point to a reverse link, suggesting that the intensity of COVID-19 symptoms might have a long-lasting detrimental influence on anxiety and depression [58]. Given the complex interplay of factors, it is plausible that although anxiety related to finances and psychology may contribute to the pandemic experience as a whole, their correlation with COVID-19 symptoms may differ and be complicated.

In terms of education, we observed that people with higher education levels were more likely to develop symptoms of COVID-19 compared to those with lower education levels. This result may reflect differences in exposure patterns and awareness of preventive measures between different educational groups. Higher-educated people are generally more likely to recognize and report COVID-19 symptoms due to their better health literacy, awareness, and access to information, which may lead to an increased likelihood of identifying themselves as symptomatic. Conversely, individuals with lower education levels may underreport symptoms due to limited knowledge about the disease or reduced healthcare-seeking behaviour [59], [60]. Similar results were found in several studies from different regions, such as in Portugal [61] and China [62]. Also, a few studies identified the opposite result, which was that uneducated or less-educated people were more vulnerable to developing COVID-19 symptoms due to greater occupational exposure, poorer adherence to preventive measures, and limited access to healthcare [63], [64]. These contrasting findings highlight the complex relationship between education level and COVID-19 symptom development, which may be influenced by cultural, economic, and social factors in different regions.

The study also observed that chronic illness is a significant risk factor for developing COVID-19 symptoms. People who already had chronic diseases had a higher chance of developing COVID-19 symptoms. The reason behind this association may be that the immune system of participants with chronic diseases was already damaged, and, as a result, it makes people more susceptible to infections, including viral infections like COVID-19 [65], [66]. These findings were consistent with previous research that had also recognized chronic diseases as a risk factor for COVID-19 development [67], [68], [69], [70], [71].

This study also found that the development of COVID-19 symptoms differs by area of residence. The urban population of Sylhet was more vulnerable to developing symptoms than the rural population. A key reason could be that urban areas are more crowded and polluted than rural areas [72], [73]. As a consequence, COVID-19 spreads more rapidly and is deadly in the urban region. Therefore, this finding highlights the potential influence of population density and community transmission dynamics on COVID-19 infection rates [74], [75]. Many researchers found equivalent results that respondents from urban areas had a higher risk of developing COVID-19 symptoms [25], [76], [77].

### Strengths and limitations of this study

The main limitation of our study was the nature of the study design. We obtained data through retrospective interviews, and this nature of the data may have introduced recall biases. Longitudinal research would have been conducted to profile study participants over time. Also, the findings cannot be generalized for the country as we only considered the Sylhet region of Bangladesh. Despite these constraints, methodologically, it was a very strong study. We appropriately measured the intensity/structure of COVID-related anxiety. Additionally, we use a proper modeling approach to determine risk factors for the emergence of COVID-19 symptoms. Future research should replicate the findings using a nationally representative survey. It will be more significant if future researchers can conduct a longitudinal study.

### Recommendations

As the study reveals that participants with chronic diseases are vulnerable to the development of symptoms of COVID-19, they need to be more aware of this kind of pandemic in the future. We must take extra care of those members of our family who have chronic diseases. The government should emphasize taking care of those people and prioritize them in accessing treatments and vaccinations. Urban people should be more careful during the pandemic of emerging infectious diseases since the study identified that respondents in urban areas were more prone to developing symptoms. The government should take appropriate steps to rapidly protect urban residents during a pandemic and impose strict rules to reduce the risk of exposure to infectious diseases in urban areas. Furthermore, the appearance of COVID-19 symptoms was largely caused by psychosomatic anxiety. So, during the pandemic or epidemic of any emerging infectious disease, the national health authority of a country may organize some counseling sessions (either offline or online) to help psychosomatic patients reduce their false feelings of developing disease symptoms. However, it is a hugely beneficial realization that taking excessive personal and social protective measures during a pandemic/epidemic of an infectious disease acts as a buffer against the development of disease symptoms. As a final point, our study has identified the MC19P-SE scale as a well-validated and reliable tool that can be used by health professionals or researchers to assess the coronaphobia or degree of anxiety of other emerging infectious diseases of a person, especially if they intend to conduct a similar study in any region of Bangladesh or across the country.

## Conclusion

The study reveals that in the Sylhet region of Bangladesh, there is a significant association between the emergence of COVID-19 symptoms and anxiety associated with the virus. Furthermore, the results of this investigation support the notion that the MC19P-SE scale has potential validity and reliability characteristics.

## Funding

This research was funded by the SUST Research Centre, 10.13039/501100007944Shahjalal University of Science and Technology, Sylhet-3114, Bangladesh [grant number: PS/2021/1/32].

## CRediT authorship contribution statement

**Luthful Alahi Kawsar, Md. Atiqul Islam, Mohammad Romel Bhuia**: Conceptualization, Investigation, Methodology, Writing − review & editing. **Luthful Alahi Kawsar, Syed Toukir Ahmed Noor**: Data curation, Validation, Writing − original draft. **Syed Toukir Ahmed Noor**: Formal analysis. **Luthful Alahi Kawsar, Mohammad Romel Bhuia**: Supervision. All authors have read and agreed to the published version of the manuscript.

## Declaration of Competing Interest

The authors declare that they have no known competing financial interests or personal relationships that could have appeared to influence the work reported in this paper.

## Data Availability

Data is available on request.

## Footnotes

Appendix A Supplementary data associated with this article can be found in the online version at doi:10.1016/j.xjmad.2025.100108.
