# Supplementary material for: Validation of modified COVID-19 Phobia Scale (MC19P-SE) to examine the relationships between corona anxiety and COVID-19 symptoms: A case-control study
Source: J Mood Anxiety Disord. 2025 Jan 16;9:100108. doi: 10.1016/j.xjmad.2025.100108 (PMC12244174; doi:10.1016/j.xjmad.2025.100108)
Supplement: Supplementary file 1 — Supplementary material [file mmc1.docx]

**Appendix**

Appendix Table 1: Rotated factor matrix of MC19P-SE items.

| **Items** | **Communalities** | **Psychological** | **Psycho-somatic** | **Economic** | **Social/excessive protective** |
| --- | --- | --- | --- | --- | --- |
| Psy1 | 0.654 | 0.736 |  |  |  |
| Psy2 | 0.546 | 0.693 |  |  |  |
| Psy3 | 0.567 | 0.712 |  |  |  |
| Psy4 | 0.553 | 0.681 |  |  |  |
| Psy5 | 0.642 | 0.746 |  |  |  |
| Psy6 | 0.180 | 0.339 |  |  |  |
| Som1 | 0.534 |  | 0.713 |  |  |
| Som2 | 0.642 |  | 0.792 |  |  |
| Som3 | 0.688 |  | 0.802 |  |  |
| Som4 | 0.542 |  | 0.643 |  |  |
| Som5 | 0.468 |  | 0.583 |  |  |
| Eco1 | 0.528 |  |  | 0.665 |  |
| Eco2 | 0.324 |  |  | 0.476 |  |
| Eco3 | 0.417 |  |  | 0.635 |  |
| Eco4 | 0.554 |  |  | 0.644 |  |
| Soc1 | 0.641 |  |  |  | 0.738 |
| Soc2 | 0.542 |  |  |  | 0.680 |
| Soc3 | 0.444 |  |  |  | 0.567 |
| Soc4 | 0.339 |  |  |  | 0.432 |
| Soc5 | 0.480 |  |  |  | 0.483 |
| Expro1 | 0.438 |  |  |  | 0.466 |
| Expro2 | 0.275 |  |  |  | 0.429 |
| Expro3 | 0.403 |  |  |  | 0.505 |


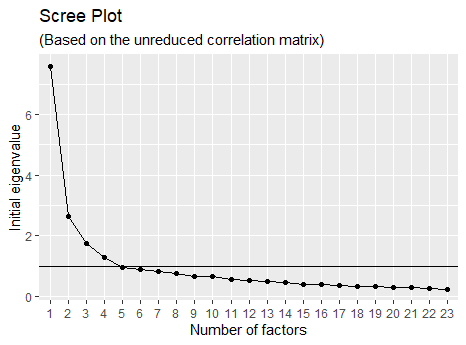


Appendix figure 1: Scree plot of MC19P-SE items.

**
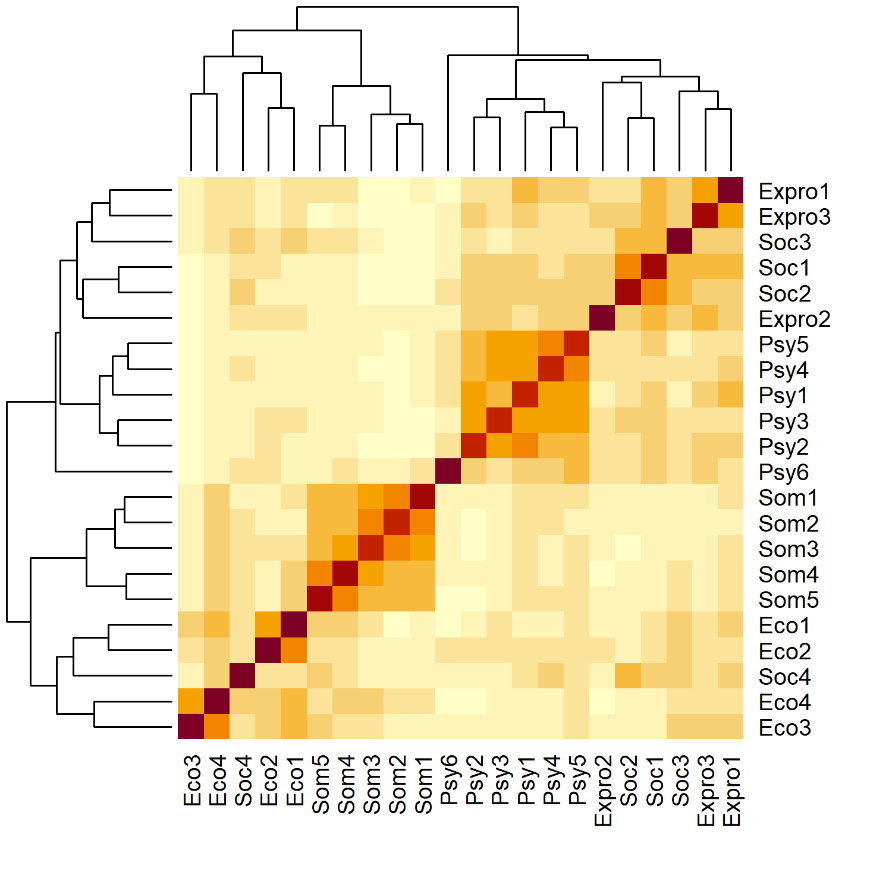
**

Appendix figure 2: Heat map of item-to-item correlation with dendrogram.


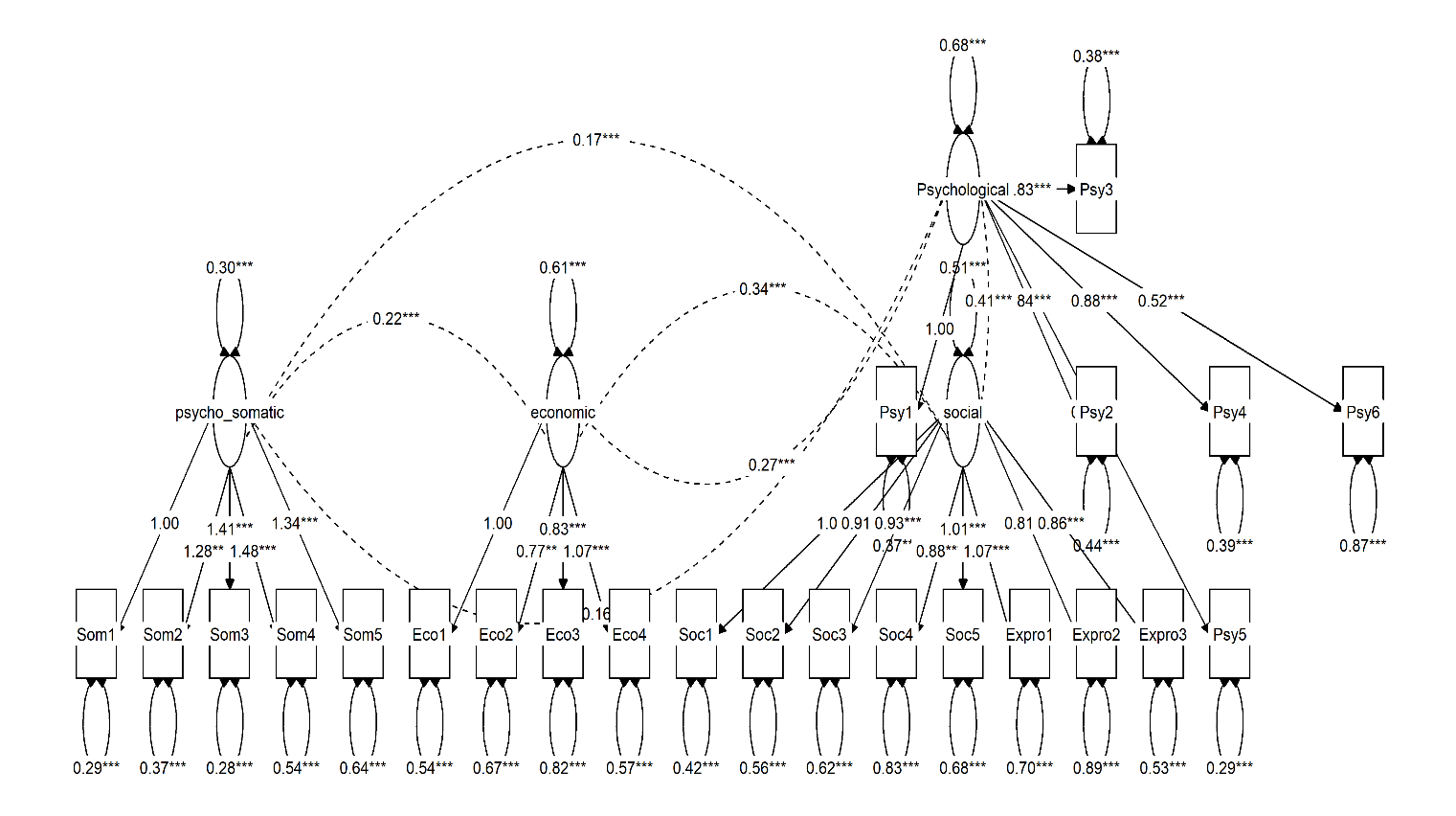


Appendix figure 3: Details of the Confirmatory factor analysis model.
